# A Wearable Multimodal Sensing System for Tracking Changes in Pulmonary Fluid Status, Lung Sounds, and Respiratory Markers

**DOI:** 10.3390/s22031130

**Published:** 2022-02-02

**Authors:** Jesus Antonio Sanchez-Perez, John A. Berkebile, Brandi N. Nevius, Goktug C. Ozmen, Christopher J. Nichols, Venu G. Ganti, Samer A. Mabrouk, Gari D. Clifford, Rishikesan Kamaleswaran, David W. Wright, Omer T. Inan

**Affiliations:** 1 School of Electrical and Computer Engineering, Georgia Institute of Technology, Atlanta, GA 30313, USA; jaberkebile@gatech.edu (J.A.B.); goktug@gatech.edu (G.C.O.); samer92@gatech.edu (S.A.M.); omer.inan@ece.gatech.edu (O.T.I.); 2 Woodruff School of Mechanical Engineering, Georgia Institute of Technology, Atlanta, GA 30332, USA; bnevius3@gatech.edu; 3 Wallace H. Coulter Department of Biomedical Engineering, Georgia Institute of Technology, Emory University, Atlanta, GA 30332, USA; cnichols46@gatech.edu (C.J.N.); gari@gatech.edu (G.D.C.); rkamales@dbmi.emory.edu (R.K.); 4 Bioengineering Graduate Program, Georgia Institute of Technology, Atlanta, GA 30332, USA; vganti6@gatech.edu; 5 Department of Biomedical Informatics, Emory University, Atlanta, GA 30332, USA; 6 Department of Emergency Medicine, Emory University, Atlanta, GA 30332, USA; dwwrigh@emory.edu

**Keywords:** wearable sensing, lung sounds, impedance pneumography, bioimpedance spectroscopy, cardiorespiratory monitoring, fluid status, heart failure, sensor fusion

## Abstract

Heart failure (HF) exacerbations, characterized by pulmonary congestion and breathlessness, require frequent hospitalizations, often resulting in poor outcomes. Current methods for tracking lung fluid and respiratory distress are unable to produce continuous, holistic measures of cardiopulmonary health. We present a multimodal sensing system that captures bioimpedance spectroscopy (BIS), multi-channel lung sounds from four contact microphones, multi-frequency impedance pneumography (IP), temperature, and kinematics to track changes in cardiopulmonary status. We first validated the system on healthy subjects (*n* = 10) and then conducted a feasibility study on patients (*n* = 14) with HF in clinical settings. Three measurements were taken throughout the course of hospitalization, and parameters relevant to lung fluid status—the ratio of the resistances at 5 kHz to those at 150 kHz (*K*)—and respiratory timings (e.g., respiratory rate) were extracted. We found a statistically significant increase in *K* (*p* < 0.05) from admission to discharge and observed respiratory timings in physiologically plausible ranges. The IP-derived respiratory signals and lung sounds were sensitive enough to detect abnormal respiratory patterns (Cheyne–Stokes) and inspiratory crackles from patient recordings, respectively. We demonstrated that the proposed system is suitable for detecting changes in pulmonary fluid status and capturing high-quality respiratory signals and lung sounds in a clinical setting.

## 1. Introduction

Heart failure (HF), which affects over 6 million Americans, imposes a significant burden on patients and healthcare systems due to the more than 1 million hospitalizations per year [1]. Acute decompensated HF, typified by the presence of pulmonary edema, dyspnea, and abnormal lung sounds [2], frequently results in hospitalizations that are associated with increased mortality [3]. Though medical treatment can effectively mitigate symptoms of decompensation, approximately 40% of discharged patients still show signs and symptoms of HF [4]. Therefore, systems that enable efficient tracking of cardiopulmonary status throughout the course of hospitalization could alert clinicians to worsening or improvement, allow for more aggressive management, and reduce the duration of hospitalization.

Existing techniques for evaluating cardiopulmonary status usually consist of single-point measurements, such as manual lung auscultation or radiographic imaging, which only supply qualitative metrics regarding a patient’s health [5,6]. The absence of practical methods for tracking pulmonary edema and extracting respiratory waveforms, typically measured with obtrusive spirometers or face masks [7], likewise presents a considerable challenge for quantifying respiratory deterioration. Therefore, wearable systems that can conveniently and continually track physiological parameters relevant to cardiopulmonary health are promising adjuncts to conventional methods. Specifically, a multimodal system that monitors fluid status through bioimpedance spectroscopy (BIS), lung acoustics via multi-location digital auscultation, and respiratory activity using multi-frequency impedance pneumography (IP) could enable comprehensive tracking of cardiopulmonary function.

Prior work has illustrated the efficacy of these individual modalities for capturing salient cardiopulmonary health markers. BIS has previously been employed in the detection of pulmonary fluid accumulation in patients with HF [8,9]. Similarly, digital lung auscultation enables the quantification of acoustic properties that have long been used to distinguish cardiopulmonary disorders [10,11], and promising results have recently been reported from single- [12] and multi-channel lung sounds recordings [13,14]. Lastly, IP has been used to accurately estimate respiratory parameters which have shown relevance for assessing pulmonary function [15,16] and evaluating the risk of breathing disorders [17]. Though these signals have demonstrated utility when used independently, they are commonly measured with expensive benchtop equipment that precludes expediency and extended assessments. Only recently has work been dedicated to the development of systems suitable for multimodal cardiopulmonary monitoring, but none have been tested in clinical populations [18,19,20]. Moreover, the fusion of these cardiopulmonary sensing modalities and their resulting clinical impact have been sparsely investigated. Therefore, the role and efficacy of multimodal wearables for cardiopulmonary sensing warrants further investigation, particularly in patients with respiratory distress such as HF, COVID-19, pneumonia, or acute respiratory distress syndrome (ARDS). 

In this work, we present a novel wearable multimodal sensing system for capturing simultaneous lung sounds from four sites on the anterior and posterior sides of the chest, IP-derived respiratory waveforms, and BIS-based fluid measurements to assess cardiopulmonary health status, with concurrent kinematic and temperature data. We first validated the system in 10 healthy subjects, demonstrating the ability to acquire high-quality signals. Then, we conducted an in-clinic study on 14 patients with HF and acquired three measurements during hospitalization, including admission and discharge, to evaluate the feasibility of our system for tracking changes in pulmonary fluid distribution and extracting pertinent respiratory markers and sounds. By addressing the scarcity of practical cardiopulmonary monitoring techniques, the system proposed in this work can potentially equip clinicians with valuable indicators of cardiopulmonary health that lead to better titration of care and improved patient outcomes.

## 2. Materials and Methods

### 2.1. System Adaptation for Respiratory Sensing

We adapted our joint health monitoring multimodal sensing system [21] to suit wearable respiratory sensing in clinical settings. Briefly, the system is composed of two main subsystems: the audio and main boards. The audio board samples data from four audio channels simultaneously, while the main board records data from two inertial measurement units (IMUs), two temperature sensors (Temps), and an electrical bioimpedance (EBI) front-end. The system can operate in either continuous or spectroscopy mode, each uniquely capturing pertinent physiological information, which determines what sensors are active and the sampling scheme that is employed. The following sections address the updates and modifications made to the system.

#### 2.1.1. Hardware Modifications 

To enable lung sounds measurements, which are commonly believed to cover the frequency range from 50 Hz to 2500 Hz [2], we modified the analog front-end (AFE) of the audio board to decrease the filter’s lower cut-off frequency to have sufficiently wide bandwidth (32 Hz–20 kHz). The rest of the audio board specifications remained unchanged from the previous version [21].

The impedance portion of the main board, originally designed to measure EBI of joints, was refined in [16] to measure transthoracic EBI. The AFE was replaced with the AD5940 integrated circuit (Analog Devices, Cambridge, MA, USA). This integrated circuit generates a sinusoidal 450 mV_peak_ excitation signal, constrained by current-limiting resistors to meet the IEC 60601-1-11 safety guidelines [22], and measures the delivered current and resulting voltage drop across the tissue with a four-electrode configuration using standard 3M electrodes (3M, St. Paul, MN, USA). For each current and voltage measurement, 18-bit real and imaginary values are returned, which can be calibrated [23] to form a complex impedance. The operational modes, detailed further in Section 2.1.5, determine the EBI measurement parameters, such as excitation frequency, and the resulting sampling rate. Table 1 summarizes the electrical specifications of the updated main board. 

#### 2.1.2. Packaging Design

**Figure 1:**
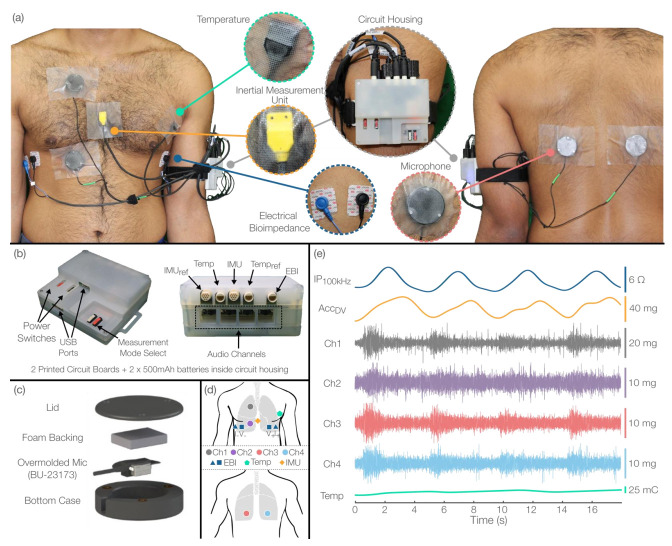
(**a**) Overview of the redesigned system for respiratory monitoring. A central circuit housing connects to each sensor independently and can be attached to the arm via Velcro strap. The semi-transparent material of the box provides visual feedback through LEDs. The system contains four audio channels, 2 IMUs (1 reference), 2 temperature sensors (1 reference), and 2 pairs of EBI electrode wires. (**b**) Central circuit housing hosting the audio and main boards PCBs, 2 500 mAh batteries, and mechanical switches to initiate/stop recordings and control the operation mode. All connectors enter the box from the same side via right-angle connectors. (**c**) Custom 3D-printed microphone case to provide constant backing force. The contact microphones (BU-23173-000, Knowles Electronics LLC., Itasca, IL, USA) were professionally overmolded in a 77 A durometer silicone. (**d**) Placement of multimodal sensors utilized in this work. (**e**) Exemplary recording from a selected subset of sensors during a deep breathing maneuver.

The redesigned form factor consists of a central circuit housing (7.90 × 6.92 × 3.52 cm, 122 g), professionally injection-molded with thermoplastic polylactic acid (PLA) material (Proto Labs, Maple Plain, MN, USA), that connects to each sensor independently (Figure 1a). The housing was fabricated in a semi-transparent material to provide visual feedback to users through LEDs and provide support for attachment to the arm or clothing via Velcro strap or metal clip, respectively. As in the previous version, the circuit housing hosts the main and audio boards, a pair of 500 mAh batteries, and mechanical switches to both initiate/stop recording and control the operation mode. In this work, we reoriented the PCBs and incorporated right-angle connectors so that all connections enter the circuit housing from the same side and at the same angle (Figure 1b). Such arrangement improves cable management and organization and facilitates connection to the sensors. 

To allow for modular placement of sensors, we detached the sensors connected to the main board from their previous cases, resulting in independent pairs of sensors (IMU and Temp) and EBI snap electrode cables. For both the IMUs and Temps, a single sensor was left for measurement directly on the skin, and the remaining one was placed in the circuit housing for reference measurements. The sensors in direct contact with the skin were professionally overmolded and assembled (Winchester Interconnect, Norwalk, CT, USA) to provide sealing. This encasement not only prevents the skin from interacting with circuit components, but also moisture and sweat from damaging the sensors, allowing for sanitization. The reference sensors, on the other hand, were protected by enclosing them in custom 3D-printed flexible cases utilizing a 95 A Cheetah 3D printer filament (Cheetah 95 A TPU, NinjaTek, Manheim, PA, USA) and then secured with epoxy.

#### 2.1.3. Acoustic Design

We developed a new microphone attachment solution with the previously used low-noise and wide-bandwidth contact microphones (BU-23173-000, Knowles Electronics LLC., Itasca, IL, USA) at its core. Our group previously compared air microphones and contact microphones for the purpose of knee sounds measurements, and we showed that air microphones are more prone to background noise and that the acoustic energy attenuation through air reduces the signal-to-noise ratio [24]. For lung sounds measurement, when an air layer is used as the propagation medium between a diaphragm and the acoustic sensor (e.g., electret microphone), signal degradation is also expected due to their high sensitivity to environmental sounds [25]. Therefore, contact microphones—such as the ones used in this work—are inherently less sensitive to environmental sounds than their air-coupled counterparts because they couple directly to skin.

To provide solder joint protection and water-proofing capabilities, necessary qualities to deploy the system in uncontrolled clinical settings, the contact microphones were professionally overmolded in a 77-shore hardness (A-scale) durometer silicone (Winchester Interconnect, Norwalk, CT, USA). In prior work [26], we found this durometer to be appropriate for preserving linearity and ensuring a bandwidth within the frequency range of interest for lung sounds. While adding this silicone layer between the sensor and skin may result in acoustic energy reflection due to the acoustic impedance mismatch of the boundaries, silicone rubber has been utilized as an acoustic conduction material in custom auscultation devices due to having an acoustic impedance that is much greater than that of air but similar to the acoustic impedance of skin, thereby minimizing ambient noise coupling and maximizing vibration transmission [25,27]. Thus, the overmolded contact microphones utilized in this work provided virtual direct coupling to skin and acoustic isolation from airborne sounds.

While the overmolded microphones provide a convenient and robust way of measuring lung sounds with high quality, uncontrolled backing force on the contact microphones has recently been shown to change the frequency response of a contact microphone significantly [28] and is not recommended for measuring lung sounds with contact-type acoustic sensors [29]. Given the potential application of our system to people in bed, possibly in a supine posture, a well-controlled backing force was necessary to standardize the acoustic measurements. To address this, we designed a custom rigid enclosure, 3D-printed in PLA material, to provide constant backing force. The custom cases (3.97 × 3.97 × 1.37 cm) consist of a base layer, containing a slit that exposes the microphone sensing surface, and a lid that is secured via three screws (Figure 1c). The overmolded microphones were inserted into the base case, and then the remaining cavity was filled with non-conductive foam to generate a constant force pushing the microphones towards the skin when the lid is secured. To check the effects of the designed case on the transfer function of the overmolded microphones, we conducted shaker vibration testing following the procedure described in [28] and found no significant differences (Figure S1).

Note that we decided not to use any adhesives between the contact microphone and the skin, as prior work showed that they alter the frequency response of the microphone [28]. Instead, we used 3M Transpore surgical tape (3M, St. Paul, MN, USA) to effectively secure the microphone casings against the skin (Figure 1a). Several adhesion alternatives were evaluated, and we found this tape to provide the best adhesion to various skin surfaces, especially on hairy and sweaty skin.

#### 2.1.4. Sensor Placement

Figure 1d shows the sensor placements used in this work. The EBI electrode pairs, each consisting of one voltage and one current electrode, were placed on opposite portions of the thorax at the intersection of the midaxillary and 5th intercostal line, all lying in the same horizontal plane. The voltage electrodes were positioned anterior relative to the current electrodes, separated by an approximated distance of 5 cm. The resistive component of the EBI signals measured from similar electrode configurations has formerly exhibited strong linearity with lung volume [30] and has also been employed in prior thoracic BIS studies [31]. To acquire multi-channel lung sounds data, we placed the microphones over the anterior right upper and lower chest quadrants (CH1-2) and posterior left and right lower chest quadrants (CH3-4). This placement follows the computerized respiratory sound analysis (CORSA) recommendations for multi-channel lung auscultation [32]. The temperature sensor was placed close to the armpit, providing surface skin temperature readings. Additionally, while the IMU was initially designed to provide postural and kinematic information relative to the reference sensor, we placed the IMU on the xiphoid process to also capture respiratory-related chest movements. Although multiple sensors and wires were placed around the chest due to the multimodal nature of the system, the wires were long enough to allow unrestricted natural breathing motion. Figure 1e shows the recordings of a selected subset of sensors during a deep breathing maneuver.

#### 2.1.5. System Operational Modes

For this work, firmware was developed to enable the main board to alternate between two operational modes: (1) spectroscopy mode, measuring BIS across a logarithmically distributed range of 32 excitation frequencies from 5 to 150 kHz, and (2) continuous mode, concurrently capturing the multi-frequency IP signal at four frequencies (5, 50, 100, 150 kHz). The BIS excitation frequencies were selected to encapsulate the distinctive tissue impedance properties at low and high frequencies, which have previously been employed to assess pulmonary fluid status [33,34], whereas the excitation frequencies for continuous IP measurements were selected in accordance with prior work from our group, which demonstrated strong correlations to respiratory waveforms acquired from gold-standard methods [16]. The system can perform a sweep of the 32 frequencies every 2 s or sample an individual frequency every ~15.6 ms, dictating a sample rate of 16 Hz for each of the four excitation frequencies in the continuous measurement mode. When continuous mode on the main board is active, simultaneous measurements are acquired from both IMUs (100 Hz), both Temp sensors (1 Hz), the EBI front-end (16 Hz × 4), and the four microphones (46.875 kHz × 4) from the audio board. Alternatively, spectroscopy mode samples only the main board’s sensors, using a different EBI sampling scheme (1 sweep/2 s), with the audio board remaining idle. Data collected from either operational mode are stored onto an SD card, where they can be retrieved via a USB interface for subsequent analysis. If no measurement is active, the system resides in a low-power sleep mode to preserve battery life. Table 1 details the differing current consumption characteristics of these modes. 

### 2.2. In-Lab Validation of System

We obtained approval from the Georgia Institute of Technology Institutional Review Board (IRB) to evaluate the system on human research participants (H20329). Written consent was obtained from 10 (6 males, 4 females) young and healthy volunteers (age: 25.30 ± 2.41 years, weight: 73.83 ± 15.39 kg, height: 175.31 ± 14.07 cm) with no history of cardiopulmonary disorders.

To validate our system against a ground truth auscultation device, a commercial digital stethoscope (Eko CORE, Eko Devices, Oakland, CA, USA) in diaphragm mode was placed next to Ch3 (Figure 1d), and both systems recorded simultaneously while the participants took deep breaths over a 30 second period. The participants were in sitting position, and a nose clip was used to enforce mouth breathing [32]. The average lung sound spectra from both systems were calculated across all subjects and plotted together. Additionally, three spectral features were extracted from each spectrum, namely the spectral peak (Fmax) and the frequencies that contain 50% (F50) and 95% (F95) of total the spectral power. This analysis was carried out in MATLAB R2020b (MathWorks, Natick, MA, USA).

The validation of the IP signals acquired with our system as respiratory surrogates was demonstrated in previous work [16], where we found that these IP-derived respiratory signals are highly correlated to tidal volume (TV) and can accurately estimate respiratory timings. The BIS measurement accuracy was evaluated with known impedances, which were designed to be in the same range of values as common thoracic impedances, and the corresponding errors are reported in Table 1. 

### 2.3. Proof-of-Concept Clinical Study

After validating the system on healthy controls, we obtained approval from the Emory University School of Medicine IRB (IRB00000794) and the Grady Oversight Committee to collect data from 14 (9 males, 5 females) patients with HF. The population demographics are detailed in Table 2. All patients (*n* = 14) provided verbal consent prior to data collection, which was conducted entirely at Grady Memorial Hospital in Atlanta, GA. Patients were excluded in the presence of other substantial comorbidities, such as chronic asthma, chronic obstructive pulmonary disorder (COPD), major lung injury, or history of chronic tobacco or marijuana use, or if the patient had an implantable cardioverter defibrillator (ICD).

Patients were recruited near admission with the intention of collecting three measurements across the duration of their hospitalization: within 24 h of admission, 24 h after the initial measurement, and finally within 12 h prior to discharge. Data collection times were distributed as such to allow patient treatment, particularly the prescribed diuretics, to decrease intraparenchymal fluid levels and improve respiratory status [2]. The device was placed as shown in Figure 1a,d for each measurement, which consisted of one minute of the previously described spectroscopy mode and seven minutes of continuous mode measurement. Prior to the start of recordings, the patients were told to stay in the posture most comfortable for them. To maintain consistency across all recordings, the patients were asked to remain in the same posture as the first day. Additionally, to capture a representative depiction of the patient’s health status, no instructions were provided regarding the rate and depth of breathing. Following the completion of the protocol, the system was removed and sanitized. The duration of the measurements was selected to ensure an ample number of breaths were recorded for extracting respiratory parameters while also minimizing the impact on the typical workflow of the hospital. If patients were unable or chose not to continue in the study, then no further measurements were taken. In total, 32 measurements were collected from the 14 patients: three measurements from eight subjects, two measurements from two subjects, and one measurement from four subjects.

### 2.4. Signal Visualization and Interpretation

The continuous multimodal data from the proof-of-concept clinical study were plotted simultaneously to observe the multi-channel lung sounds as a function of their location (Ch1-4) and their associated respiratory phases (inspiration and expiration) provided by the IP-derived respiratory signal. Prior to visualization in time and time–frequency domains (spectrogram), lung sounds measurements were resampled to 6 kHz using a finite impulse response (FIR) antialiasing low-pass filter. Then, the signals were filtered using a zero-phase minimum-order FIR band-pass filter in the passband 100 Hz to 1000 Hz. These frequency cut-offs were selected to suppress heart sounds interference below 100 Hz [29] and hospital room noises (e.g., alarms, television, speech) that were clearly heard above 1.8 kHz due to relatively low acoustic power of lung sounds above that frequency. To calculate the spectrograms, short-time Fourier transform (STFT) was employed with 300 ms windows and 95% overlap. These STFT windowing parameters were selected experimentally to improve the quality of the time–frequency visualizations since a wide range of values have been reported in the literature, including 500 ms windows with 50% overlap [35] and widow sizes of 16, 32, 64, 128, 256, and 512 ms with 75% overlap [36]. Instances of abnormal breathing patterns and lung sounds found in the proof-of-concept recordings are presented, and a corresponding interpretation is provided. The accelerometer data were pre-processed following the steps in Section 2.5.1.

### 2.5. Signal Processing

#### 2.5.1. Continuous Data

A signal processing pipeline was developed in MATLAB R2020b (MathWorks, Natick, MA, USA) to process the continuous data. In this work, only the 100 kHz IP signal was processed due to its high signal quality as well as being a commonly used IP frequency, although the same steps apply to any of the IP signals. 

**Figure 2:**
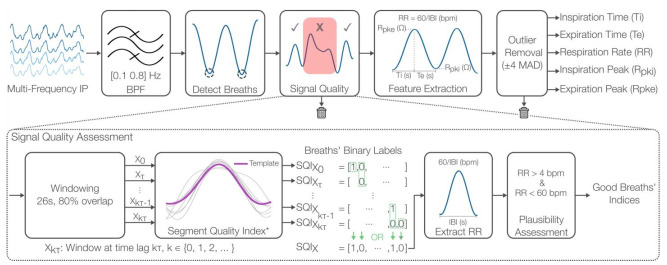
Multi-frequency impedance pneumography (IP) signal processing pipeline. After filtering the signals, breaths are detected, and their signal quality is assessed. This assessment employs overlapping windows to enable breath-by-breath evaluations with the SQI published in (Charlton et al., 2021) at its core. A final stage of plausibility assessment ensures that only breaths yielding physiologically plausible respiratory rates (RR) are deemed as good quality. These good breaths are then used to extract amplitude (Rpki/e) and timing features (Ti/e, RR). Outliers are finally removed if any of the features lay outside ±4 median absolute deviations (MAD) from the overall median.

The raw IP signals were first linearly resampled to 100 Hz with an anti-aliasing low-pass filter. Then, the signals were filtered with a Kaiser window FIR filter in the passband 0.1 Hz to 0.8 Hz, corresponding to a respiratory rate (RR) of 6 breaths per minute (bpm) and 48 bpm, respectively (Figure 2). 

To detect breath onsets, we windowed the signals (26 s windows, 80% overlap) and then extracted valid breaths from each window following the steps in [37]. Overlapping was used to avoid missing truncated breath candidates during windowing. The identified breath onsets across all windows were fused into a unique set of onsets. An inter-breath interval (IBI) was defined as the time spanning between two valid breath onsets.

After identifying the set of IBIs, we evaluated the signal quality of the corresponding breaths based on their relative morphology and duration. First, a set of overlapping windows (26 s windows, 80% overlap) was created $\left\{X_{0},\;X_{\tau },\;\ldots ,\;X_{k\tau }\right\}$, where $\tau =\left(1-0.8\right)*26=5.2\;s$ and k={0, 1, 2, …}. Overlapping windows were used to allow breaths to appear in multiple windows. We then used a modification of the signal quality index (SQI) presented in [37] to assign binary quality labels to each window. The original SQI parameters were adjusted empirically to better suit our dataset. The parameter changes are the following: (1) the window length was reduced to 26 due to higher-than-normal RRs, (2) the minimum correlation to a breath template was set to 0.7, and (3) the coefficient of variation of breath durations was set to 0.5. The resulting binary label of a given window ($X_{k\tau })$ was then replicated to its constituent breaths $\left(SQI_{Xk\tau }\right)$ to enable a stage of breath-by-breath quality evaluations across all overlapping windows. To create the final quality labels $\left(SQI_{X}\right)$, all breaths’ labels were fused through a logical OR operation, i.e., a breath is considered “good” if it was found to be “good” in any window. As a last step in the signal quality assessment, we calculated RR as 60/IBI for all detected IBIs and assessed their physiological plausibility. “Bad” breaths were defined as those resulting in a RR lower than 4 bpm or greater than 60 bpm and thus were discarded from further processing. Visual inspection was conducted on all recordings to check the quality of the accepted breaths. 

Following quality assessment, respiratory timings and amplitude features were extracted on a breath-by-breath basis. The RR was calculated as above, while the timing features inspiration time (Ti) and expiration time (Te) were extracted as in previous work [16,38]. Further, the expiration-to-inspiration ratio (Te:Ti) was calculated to provide insight into the relative contribution of each phase to the breathing cycle, which relates to ventilation [39] and breathing regularity [38,40]. The amplitude features inspiration peak (Rpki) and expiration peak (Rpke) were also calculated as in previous work [16]. As a final stage, a simplified version of the feature-based outlier rejection approach in [38] was employed, in which we flagged values lying outside ±4 median absolute deviations (MAD) from the overall median for each feature separately and then fused them to create a single set of “good” IBIs from which the final features were computed. 

Due to the well-known dependency of IP on electrode placement [31,41] and posture [42], the estimation of lung volume markers (e.g., TV) from IP-derived amplitude features requires calibration with a ground truth spirometer signal [16,30,42]. In this work, however, the absolute amplitude features (Rpki and Rpke) were only used for the feature-based outlier rejection stage of the processing pipeline and were not utilized as lung volume markers. Since only respiratory timings (RR, Ti, and Te) were compared, the slight differences in electrode placement and posture across recordings should have no significant effect on the results. Therefore, no calibration procedure was needed.

#### 2.5.2. Spectroscopy Data

A representative BIS curve was formed by averaging all measured sweeps from a measurement session, totaling to approximately 30 individual sweeps, to negate variability due to respiration or movement. One patient with only one measurement session was not included in the BIS analysis due to an error during the data collection. For all other subjects, each measurement session resulted in a single averaged BIS curve being computed. Commonly, such BIS measurements are utilized in electrical models of tissue, such as the Cole model [43], to extract parameters including the extra- and intracellular resistance (Re and Ri). However, due to confounding factors in the acquisition of thoracic BIS measurements [44,45] which can result in positive phase errors at high frequencies [46], Cole modeling may not be an appropriate method of analysis. As an alternative, the resistance measured at low frequencies—which relates to extracellular paths—and at high frequencies—which encompasses both extra- and intracellular paths—were employed in place of the Re and Ri parameters, respectively. This method simplifies the analysis and avoids the error inherent to the extrapolation required by Cole modeling. Thus, the resistance at 5 kHz (R5k) and at 150 kHz (R150k) were extracted from the averaged BIS curve. Finally, the ratio of R5k to R150k, denoted hereafter as *K*, was computed to provide meaningful insight into the distribution and accumulation of fluid between extra- and intracellular spaces, which is often exacerbated in patients with HF [34]. Comparable whole body BIS methods employ similar rationale when comparing extracellular water (ECW) to total body water (TBW) to detect fluctuations in fluid status for patients with HF [47]. While BIS measurements are sensitive to changes in posture and dependent upon consistent electrode placement, the reproducible electrode configuration employed in this study allowed for consistency across recordings. Inter-subject variability also heavily influences the raw resistance values measured during BIS, as discussed in Section 2.5.1, but our analysis normalized the measurements when taking the ratio of low to high frequencies, thus mitigating the differences in patients’ thoracic resistances and allowing for equivalent comparisons to be made.

### 2.6. Statistical Analysis

Statistical analysis was carried out in Python and MATLAB R2020b (MathWorks, Natick, MA, USA) to evaluate differences between the admission and discharge data. The two data groups were first tested for normality with the Shapiro–Wilks test [48]. If both groups were normally distributed, two-tailed paired t-tests were utilized; otherwise, the Wilcoxon signed-rank test was performed where the assumption of normality was not upheld. A *p*-value lower than 0.05 was considered statistically significant for this work.

## 3. Results and Discussion

### 3.1. Acoustic Validation against Eko CORE

**Figure 3:**
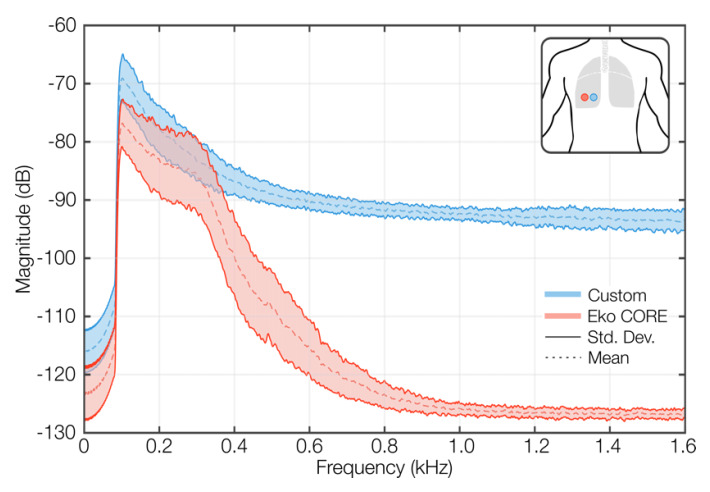
Validation against the Eko CORE digital stethoscope. The mean and standard deviations for both spectra are plotted. The data were obtained from 10 healthy volunteers with both sensors close to each other and over the posterior left lower chest quadrant. Both systems recorded simultaneously while the participants took deep breaths over a 30–s period in sitting position.

The results from the acoustic validation against the Eko CORE digital stethoscope are illustrated in Figure 3 and Table 3. The spectral comparison in Figure 3 shows that both spectra peak around the same frequency and have similar frequency content below 400 Hz. The marked difference in spectral power above 400 Hz was anticipated due to the limited frequency range of normal lung sounds (95% of the spectral power lies below 500 Hz [49]) and the embedded noise-canceling capabilities of the Eko CORE. These observations are confirmed by the spectral features extracted from both spectra (i.e., Fmax, F50, and F95), which are reported in Table 3. The slight difference of only 4.98 Hz in Fmax corroborates the agreement between the spectral peaks of both systems, which are consistent with the spectral peak of normal lung sounds reported in the literature [49]. Similarly, the differences in F50 and particularly F95 highlight the noise-canceling capabilities of the Eko CORE and the frequency response of the stethoscope diaphragm that concentrate most of the acoustic energy around the peak frequency of normal lung sounds. This aggressive attenuation provided by the Eko CORE, which is also found in other commercial digital stethoscopes such as Thinklabs One, has been demonstrated to attenuate high-pitched sounds [50]. Thus, the increased spectral power that our system provides at higher frequencies could be particularly advantageous for capturing lung sounds with frequency content beyond the passband of conventional stethoscopes, including fine crackles (>650 Hz) and wheezes (>400 Hz) [10,51]. 

The similarities of lung sounds captured with our system and the Eko CORE were also confirmed in the time and time–frequency domains (Figure S2).

### 3.2. Results from Proof-of-Concept Clinical Recordings

#### 3.2.1. Detecting Changes in Pulmonary Fluid Status

**Figure 4:**
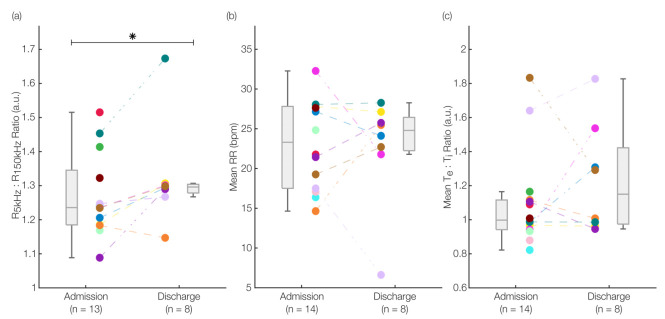
Results from proof-of-concept clinical recordings. (**a**) Differences between the admission and discharge groups for *K* (the ratio of the resistances at 5 kHz and 150 kHz) showing a statistically significant increase (*p* < 0.001, Wilcoxon signed-rank) from *K* = 1.27 ± 0.12 to *K* = 1.32 ± 0.15. This statistically significant increase in *K* indicates the reduction of pulmonary fluid or its redistribution into the appropriate intracellular compartments. (**b**) Differences in the mean RR from admission to discharge groups showing a slight decrease from 23.12 ± 5.53 bpm to 22.73 ± 6.85 bpm, not statistically significant. (**c**) Differences in the mean Te:Ti ratio showing a slight increase from 1.10 ± 0.27 to 1.23 ± 0.32, not statistically significant. * denotes a *p*-value lower than 0.05 and was considered statistically significant for this work.

Figure 4a demonstrates a statistically significant (*p* < 0.001, Wilcoxon signed-rank) increase in *K* from admission (*K* = 1.27 ± 0.12) to discharge (*K* = 1.32 ± 0.15). For the patients with both measurements available (*n* = 8), we observed an increasing trend in all but one patient. The magnitude of the average increase in *K* is consistent with previous work that examined changes in the ratio of the extra- and intracellular resistances in patients with HF and those undergoing pleural effusion [34]. In general, as the patients experience diuresis and transition to a compensated state, the overload of pulmonary fluid can be assumed to subside as per HF discharge criteria [2]. Thus, the increase in *K* suggests that this metric of fluid distribution can track the progression, and perhaps the effectiveness, of the applied treatment. For the only patient with a decreasing trend, given that they had the highest BMI (97.2 kg/m^2^) of all the patients, we hypothesize that tracking pulmonary edema specifically, as opposed to general thoracic fluid, in such patients with considerably enlarged thoraxes might require greater sensitivity or additional segmental measurements. Alternatively, this particular patient’s pulmonary edema may not have decreased from admission to discharge. However, in this study, there were no gold-standard markers of fluid volume to compare against, so specific quantification of edema cannot be made. 

As the BIS measurements encompass the entirety of the thoracic cavity, it is difficult to relate changes in EBI directly to fluid in the lungs, which is typically only possible via segmental EBI, necessitating the use of more electrodes and complex hardware [8,33]. However, variations in *K* not only indicate the flux of fluid in extracellular spaces, which is associated with edema, but also the redistribution of fluid back into the appropriate intracellular compartments [34,52]. This allows for a more sensitive measure of fluid changes and offers insight regarding the location of fluid accumulation. A secondary outcome from the use of this analysis is the lack of dependency on obtaining measurements from a large number of excitation frequencies, as only two measurements, a pair of low- and high-frequency measurements, are needed. Thus, the continuous measurement mode described in this work could also be utilized to also capture fluid status in addition to respiratory waveforms, simplifying the firmware further and reducing the amount of data required. Overall, this simplified BIS analysis could provide clinicians with indications of fluid retention and signs of acute decompensation or, alternatively, transition to a compensated state. 

#### 3.2.2. Extraction of Respiratory Health Markers

For the first time, to the best of our knowledge, we demonstrated the use of IP to capture breath-by-breath respiratory features in an HF population. Figure 4b,c show the differences in mean RR and mean Te:Ti between the admission (*n* = 14) and discharge (*n* = 8) groups. We found a slight decrease in the mean from 23.12 ± 5.53 bpm to 22.73 ± 6.85 bpm for RR and a slight increase from 1.10 ± 0.27 to 1.23 ± 0.32 for the mean Te:Ti, both statistically insignificant (*p* > 0.05, Wilcoxon signed-rank). Beyond the differences (or lack thereof) in these markers from admission to discharge, these results show that the values extracted are in physiologically plausible ranges. The mean RR was above the normal range in adults (12–20 bpm [53]), as expected in decompensated HF patients [54]. Similarly, the mean Te:Ti values close to 1:1, with some values lower than one, indicate the need for increased ventilation since a greater portion of the breathing cycle is spent in inspiration (where gas exchange occurs) as compared to the normal 2:1 ratio [39]. These results suggest that the proposed system can be utilized to extract relevant respiratory markers in a clinical setting. 

#### 3.2.3. Cheyne–Stokes Respiration (CSR)

**Figure 5:**
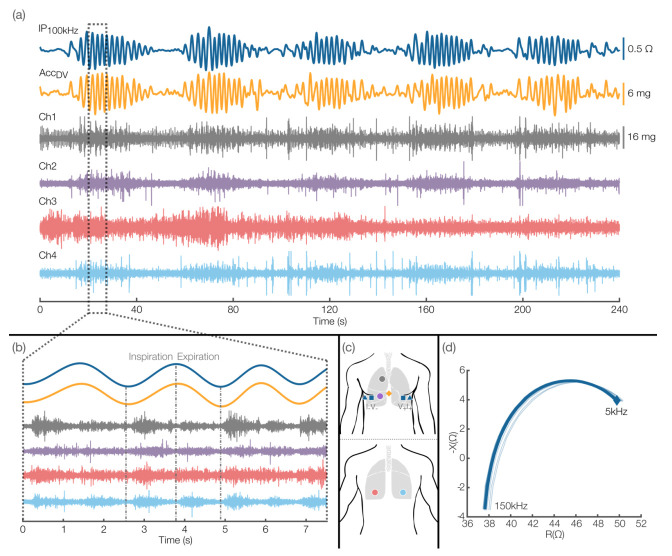
Abnormal breathing pattern finding from the proof-of-concept clinical recordings. (**a**) Segment of multimodal data obtained from patient 13, in which they were breathing following the CSR pattern. (**b**) Seven–second segment from the first CSR event in (**a**). (**c**) Placement of sensors used in the recordings. (**d**) Mean BIS curve for this patient.

A 240 s segment of multimodal data obtained from patient 13 in which they were breathing following the CSR pattern, is illustrated in Figure 5a. Simultaneously recorded IP (IP_100 kHz_), chest motion in the dorsoventral direction (ACC_DV_), and lung sounds (Ch1-4) are plotted. The sensors placement described above was used and reproduced in Figure 5c for clarity. CSR is a distinctive breathing pattern characterized by cyclic events in which the breathing goes from gradual hyperpnea (deep breathing) to gradual hypopnea (shallow breathing), bounded by apneic periods (cessation of breathing) [55]. The CSR pattern is a common finding in HF patients, associated with increased mortality that is even higher when occurring during daytime [56]. The two respiratory estimates shown in Figure 5a (IP_100 kHz_ and ACC_DV_) illustrate five CSR events, and while the 240 s scale complicates the visualization of the corresponding lung sounds activity, the increase in amplitude and seemingly spiking activity during each CSR event is an indication of the correlated activity between the respiratory estimates and the lung sounds channels. The morphological differences across channels are mainly due to the following well-known properties of lung sounds: (1) lung sounds measured over the chest wall are critically dependent on the origin of the sounds along the respiratory tract and the path that these sounds travel to reach the acoustic sensor (including primary airways, parenchyma, bones, muscle, fat tissue, or a combination of these) [57]; and (2) lung sounds are dependent on the airflow rates achieved during the recording [58]. For example, normal lung sounds have been demonstrated to have an intensity that is higher in the upper chest than in the lower, in inspiration than in expiration, and in the left side than in the right side [49]. Thus, since in this study, each channel was placed across different chest locations and the sounds were not elicited (i.e., no breathing depth or rate was enforced), we expected the lung sounds waveforms in each channel to be unique. The correlation to the respiratory activity is more evident in Ch1, 2, and 4 than in Ch3 because the audio quality of Ch3 was considerably lower compared to the other channels, suggesting that this microphone could have been weakly attached to the skin during this recording. Nevertheless, all lung sounds channels showed correlation to the respiratory signal surrogates in Figure 5b, where we show the first CSR event from Figure 5a in a higher time resolution. The amplitude changes triggered at each inspiratory and expiratory onset illustrate the quality of the multimodal data on a breath-by-breath level. The mean BIS curve for this patient is also shown in Figure 5d. These findings suggest that the multimodal system presented in this work provides sufficient signal quality to detect clinically valuable perturbations in breathing patterns while also capturing high quality lung sounds.

**Figure 6:**
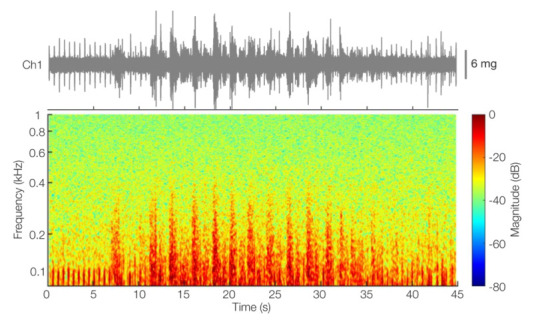
Time and time–frequency visualization of the first CSR event in Figure 5 for Ch1 (anterior right upper chest quadrant). The time–frequency visualization was obtained through STFT analysis using 300 ms windows and 95% overlap.

Furthermore, the continuous multimodal data visualization of the CSR pattern seems to illustrate the flow–sound relationship of lung sounds: as the breathing depth (and airflow) increases in each CSR event, so does the intensity of the measured sounds. In [58], this flow–sound relationship was found to be predominantly linear in healthy subjects. To gain a better insight into this mechano-acoustic behavior, in Figure 6, we visualized the time and time–frequency characteristics of the first CSR event in Figure 5. The observed frequency range of the sounds and their correspondence to the IP-derived respiration signal suggest that the sounds measured follow a flow–sound relationship, as expected. Additionally, we observed short periodic sounds during the apneic part of the CSR event, where lung sounds are absent. These are heart sounds and were expected because Ch1 was placed at the upper right chest, close to the aortic area in heart sounds auscultation [59]. Heart sounds are also present in other channels, but their acoustic energy above 100 Hz (the cut-off frequency of the high-pass filter used to mitigate heart sounds interference) will be relatively low for locations farther away from relevant cardiac auscultation points. When their relative energy is significant, they appear as low-pitched sounds of short duration, as illustrated in Figure 6. These results suggest that we could utilize a subset of the microphones to capture heart sounds as well.

#### 3.2.4. Inspiratory Crackles

**Figure 7:**
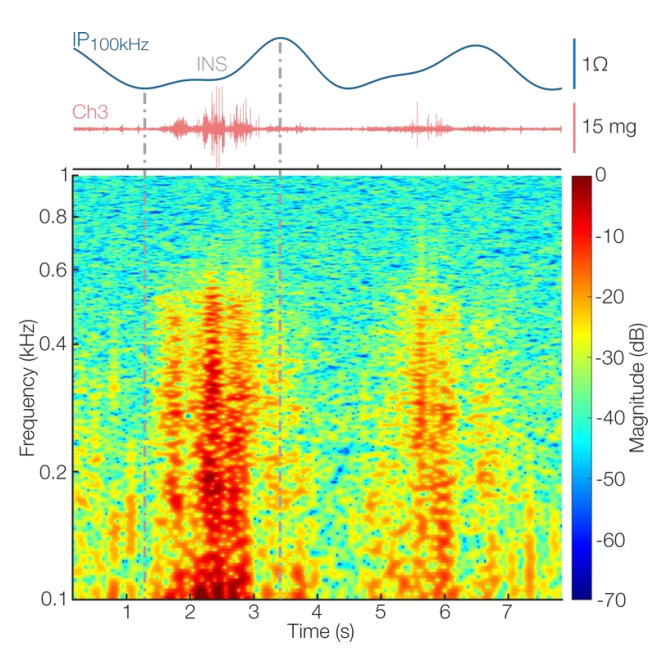
Finding of inspiratory (INS) crackles from Ch3 (posterior left lower chest) contextualized by the concurrent IP-derived respiratory signal (IP_100 kHz_) (**top**). Time (**middle**) and time–frequency (**bottom**) representations of the recorded sounds are shown. The time–frequency visualization was obtained through STFT analysis using 250 ms windows and 95% overlap.

Figure 7 shows instances of inspiratory crackles found in the posterior left lower chest quadrant (Ch3) recordings from patient 9. Both the time and time–frequency visualizations show sounds with the classical characteristics of crackles: explosive and discontinuous sounds [10,11,51]. Furthermore, crackles are commonly heard at the posterior lung bases [10], which is the case for the sounds depicted in Figure 7. While the presence of crackles is interesting on its own, the breathing context provided by the concurrent IP-derived respiratory signal greatly further enriches the finding because the specific phase in which crackles occur and the percentage of the phase occupied by them are both critical pieces of information that can help in differential diagnosis [10]. Notably, crackles during inspiration are commonly found in congestive heart failure (CHF) patients [10,51]. 

Beyond crackles, the breathing context is generally a fundamental characteristic in the description of lung sounds, and descriptions such as “strongly-inspiratory” or “Mid inspiration/expiration” are commonly found in the literature [10,51]. Therefore, we could leverage this breathing context information for the classification of a variety of lung sounds and for signal quality evaluation. During traditional lung auscultation, such contextualization occurs in real time as the sounds are elicited. For wearable auscultation systems, however, that information is only available through concurrent respiration monitoring or flow signals. To the best of our knowledge, the system proposed in this work is the first to show simultaneous high-quality respiratory data (breathing context) and multi-channel lung sounds (spatial context) in a wearable form factor.

## 4. Limitations and Future Work

### 4.1. Study Limitations

This study should be considered a demonstration of the feasibility of multimodal respiratory sensing systems due to the small cohort size. In addition, the limited amount of continuous data (7 min), which was constrained to suit the hospital’s workflow, that was acquired during each measurement session and the lack of ground truth pulmonary congestion markers make it difficult to draw explicit conclusions regarding subtle changes in respiratory activity. Longer monitoring sessions should be employed to determine whether acute changes in respiratory timings, pattern, or sounds can be detected. Furthermore, extended measurements would allow for better tracking of the fluid dynamics during the course of hospitalization.

### 4.2. Hardware Improvements

Though we demonstrated the capabilities of our system to capture insightful, high-quality multimodal data, it required a packaging solution that is not optimal for convenient, extended measurements. In order to capture all of the modalities with the proper anatomical sensor placements generalized to both low- and high-BMI patients, the resulting prototype system used lengthy cabling and a large housing for the electronics. While designing a system that is suitable for multimodal sensing across a diverse population is inherently difficult, there are several straightforward enhancements that can be made to improve the form factor and usability of the system. For instance, the microphones can be packaged in less bulky casings while still maintaining the proper bandwidth and sensitivity that is necessary for detecting lung sounds. Further, the number of microphones could be reduced. We found that Ch2 (anterior right lower chest quadrant) yielded generally low-quality recordings because it is an anatomically difficult location on which to secure the microphones with tape, particularly on high-BMI patients. Thus, a simplified system could only contain three microphones while still capturing spatially contextualized lung acoustics. Likewise, to mitigate the obtrusiveness of the EBI cables spanning across the thorax, a chest-worn electrode configuration, as used in [16], could be adopted to reduce electrode distancing and wire length. These refinements would allow for simplified sensor attachment and enable a low-profile design. To improve battery life and reduce audio processing overhead, the audio channels could be sampled at 8 kHz, which is the minimum sampling rate of the ADC used, while still having a bandwidth well beyond the lung sounds frequency range.

### 4.3. Lung Sounds Quality, Analysis, and Multimodal Fusion

Despite our efforts to mitigate the coupling of environmental sounds into the lung sounds recordings through the selection of contact microphones, the design of silicone overmolds and custom casings, and the careful selection of a robust backing tape, we found hospital room noises to be clearly audible at frequencies above 1.8 kHz. While low-pass filtering of the signals to 1000 Hz cancels these environmental noises, some lung sounds may reach frequencies above this cut-off frequency. We also found other noise sources within the frequency band of lung sounds which are not possible to remove with classic filtering approaches. Some of these noise sources could include rubbing or tapping, poor attachment to skin, and other bodily sounds. Thus, in addition to optimizing the filter parameters, in the future, we will explore signal quality assessment approaches to determine which breaths are of sufficient quality for processing. We will also explore approaches to provide stronger acoustic insulation and attachment of skin to the micro-phones.

After developing the processing pipeline to accurately extract breath onsets and confirming the correspondence between the IP-derived respiratory signal and the multi-channel lung sounds, we will now focus on fusing these signals. The IP signal will enable automatic segmentation of the lung sounds recordings into breaths (and phases) for which we can compute temporal and spectral features. These features can be used for quality assessment and for classification purposes. The breathing contextualization provided by the IP-derived respiratory signal can also be leveraged by machine learning models as a feature or label, depending on the application. Similarly, due to the change in morphology of the sounds at different chest locations, the location of the channels could be a relevant feature on its own. Furthermore, flow estimates have been accurately derived from the IP-derived respiratory signals [15] and thus could be used to study flow–sound dynamics. 

## 5. Conclusions

We have presented the validation of a novel suite of sensing modalities for assessing cardiopulmonary health status and demonstrated its feasibility for clinical deployment in a study with hospitalized patients with HF. The fidelity of the respiratory markers extracted from the clinical data indicate the ability of the multimodal system to detect changes in lung fluid levels and breathing patterns. Similarly, the multi-channel lung sounds were shown to be of sufficient quality to capture underlying changes in lung structural health. In the future, advancements in multimodal data fusion and machine learning approaches can expand the clinical relevance of our system. Furthermore, despite the marked frequency of patient readmissions due to decompensated HF [60], few studies have explored the feasibility of at-home monitoring to stratify the risk of exacerbations. Thus, future studies can assess the utility of this system, or a simplified version, for at-home daily-living pulmonary fluid status and cardiorespiratory monitoring, thereby addressing the significant gap that exists in the current state of clinical care. Beyond HF, we believe that this system also has the potential to evaluate cardiorespiratory health in patients with other respiratory complications, such as COPD, COVID-19, ARDS, and pneumonias in general. 

## Figures and Tables

**Table 1:** Main board electrical specifications.

Parameter	Value
Average Power Consumption	
Sleep	0.5 mA
Continuous Mode	27.6 mA
Spectroscopy Mode	16.8 mA
Battery Life (with 500 mAh Battery)	
Sleep	41.6 days
Continuous Mode	18 h
Spectroscopy Mode	30 h
Number of Measured Frequencies	
Continuous Mode	4
Spectroscopy Mode	32
Sampling Rate ^1^	
Continuous Mode	16 Hz
Spectroscopy Mode	0.5 Hz
Frequency Range	5–150 kHz
Excitation Voltage	450 mV_peak_
Mean Resistance (R) Error	0.50 Ω
Mean Reactance (X) Error	0.44 Ω
Noise Floor ^2^	7.8 mΩ

^1^ Sampling rate defined per measured frequency. ^2^ Computed as standard deviation of measurements of a fixed 56 Ω load.

**Table 2:** Patient demographics.

Parameter	Patient Data (*n* = 14)
Age (years), mean (SD)	50.2 (11.5)
Sex, *n* (%)	
Male	9 (64)
Female	5 (36)
Height (cm), mean (SD)	174.1 (10.1)
Weight (kg), mean (SD)	124.1 (55.3)
BMI (kg/m2), mean (SD)	41.2 (19.1)
Race, *n* (%)	
Black	13 (93)
White	1 (7)

**Table 3:** Spectral features extracted from custom system and Eko CORE digital stethoscope.

Parameter	Custom Mean (SD)	Eko CORE Mean (SD)
Fmax (Hz)	118.21 (3.50)	123.19 (9.91)
F50 (Hz)	212.67 (60.11)	150.14 (14.79)
F95 (Hz)	946.84 (170.36)	272.94 (20.09)

## Data Availability

The data presented in this study are available upon reasonable request to the corresponding author.

## Supplementary Materials

The following are available online at https://www.mdpi.com/article/10.3390/s22031130/s1, Figure S1: Transfer function comparison between plain overmolded microphones and overmolded microphones inside the custom case, Figure S2: Time–frequency visualization of a 30 s segment of lung sounds recorded with our system (top row) and the Eko CORE digital stethoscope (bottom row).
