# Supplementary material for: A Wearable Multimodal Sensing System for Tracking Changes in Pulmonary Fluid Status, Lung Sounds, and Respiratory Markers
Source: Sensors (Basel). 2022 Feb 2;22(3):1130. doi: 10.3390/s22031130 (PMC8838360; doi:10.3390/s22031130)
Supplement: Supplementary file 1 [file sensors-22-01130-s001.zip › suppl_material.pdf]

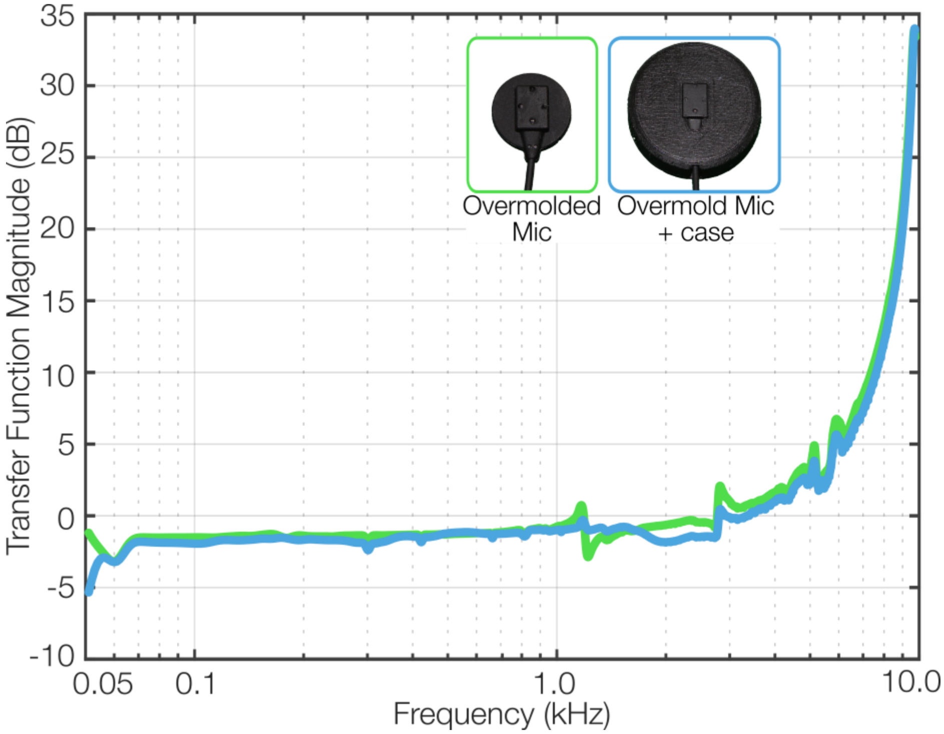

Figure S1. Transfer function comparison between plain overmolded microphones and overmolded microphones inside the custom case.

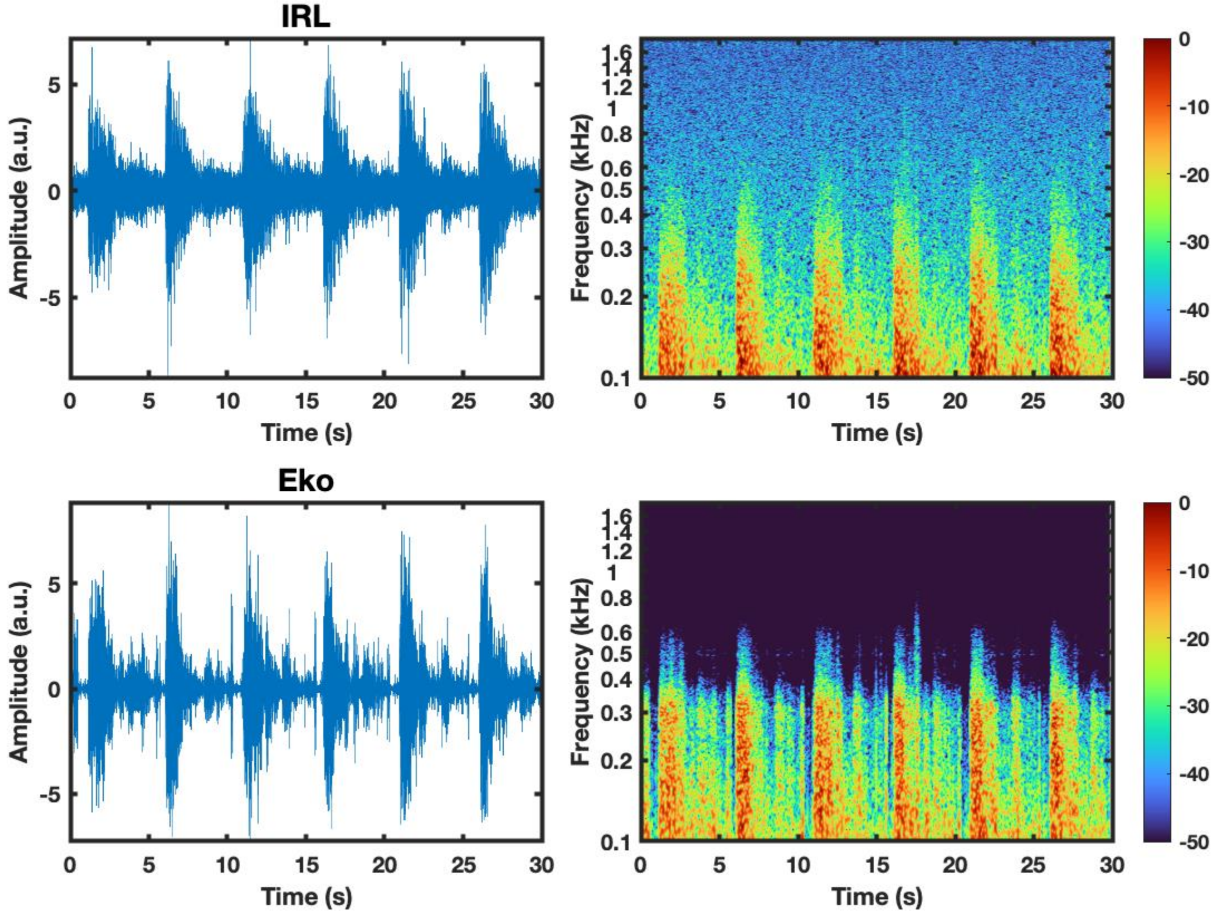

Figure S2. Time-frequency visualization of a 30-s segment of lung sounds recorded with our system (top row) and the Eko Core digital stethoscope (bottom row). The sounds were recorded from healthy volunteer at the posterior left lower chest quadrant.
